# Clinical remission following ascorbate treatment in a case of acute myeloid leukemia with mutations in *TET2* and *WT1*

**DOI:** 10.1038/s41408-019-0242-4

**Published:** 2019-10-02

**Authors:** Andrew B. Das, Purvi M. Kakadia, Damian Wojcik, Lucy Pemberton, Peter J. Browett, Stefan K. Bohlander, Margreet C. M. Vissers

**Affiliations:** 1 Centre for Free Radical Research, Department of Pathology and Biomedical Science, University of Otago, Christchurch, Christchurch, New Zealand; 2 Leukaemia & Blood Cancer Research Unit, Department of Molecular Medicine and Pathology, The University of Auckland, Auckland, New Zealand; 3 Northland Environmental Health Clinic, 2 Dip Rd, Kamo, Whangarei, New Zealand; 4 Southern Blood and Cancer Service, Southern District Health Board, Dunedin Hospital, Dunedin, New Zealand

**Keywords:** Translational research, Acute myeloid leukaemia, Acute myeloid leukaemia, Targeted therapies

Advances in DNA sequencing technologies have provided exceptional detail of the genomic and epigenomic landscapes in acute myeloid leukemia (AML)^1,2^. A key insight emerging from this data is that proteins involved in epigenetic processes are early drivers of the cancer phenotype and may be attractive drug targets^2,3^. Mutations in tet methylcytosine dioxygenase 2 (*TET2*) occur in ~10% of patients with AML^2^. TET2 is responsible for active DNA demethylation, by converting methylcytosine (5mC) to hydroxymethylcytosine (5hmC). Blast cells with *TET2* mutations from leukemia patients display increased promoter methylation and decreased hydroxymethylation compared to normal bone marrow cells, which is indicative of decreased TET2 activity^4^. Of note, mutations in *TET2* have an adverse prognostic impact on AML patients with *NPM1* mutations^5^, a group which would otherwise have a favorable outcome. This suggests that these patients do not do well with conventional treatment and may be candidates for new therapeutic approaches.

Mutations in *IDH1*, *IDH2*, and *WT1* also impact on TET2 activity^4,6^. Mutant isocitrate dehydrogenase (IDH) enzymes generate the oncometabolite 2-hydroxyglutarate, which is a competitive inhibitor of TET2^4^. Wilms tumor protein 1 (WT1) is a transcription factor that recruits TET2 to DNA, enabling promoter demethylation^7^. Notably, mutations in the *IDH1/2*-*TET2*-*WT1* pathway are largely mutually exclusive and are collectively present in 30–50% of AML cases^1,2,7^. Together, they comprise a distinct AML subtype characterized by dysregulated DNA (hydroxy)methylation (Supplementary Fig. 1).

The TET dioxygenases require O_2_, iron, 2-oxoglutarate and ascorbate for activity^8,9^ and in mouse models of leukemia with mutations in *Tet2, a*scorbate supplementation successfully restored methylation patterns^10,11^. In addition, ascorbate deficiency mimicked *Tet2* loss by cooperating with *Flt3*^*ITD*^ to promote leukemogenesis and replenishing ascorbate reversed these changes by upregulating TET2 and TET3 activity^10,12^. Ascorbate also decreased proliferation of leukemic cells with mutant *IDH1* and increased the expression of genes associated with differentiation^13^. This evidence suggests that increasing ascorbate levels could benefit patients with AML that involves decreased TET2 activity.

One recent study has investigated the use of ascorbate as an adjunct to decitabine therapy in AML patients^14^, but the effect of ascorbate for those with mutations in *IDH1/2*, *TET2*, or *WT1* is unknown. Here we report the molecular investigation of a patient with AML who failed to respond to induction chemotherapy and in whom subsequent ascorbate treatment was associated with clinical remission for over two years. We hypothesized that the observed clinical response to ascorbate treatment in this case was associated with AML driver mutations in the *IDH1/2*-*TET2*-*WT1* pathway.

This patient was diagnosed with primary AML with moderately severe pancytopenia and 75% blast cells in the bone marrow (Table 1, Fig. 1a). Granulopoiesis was markedly suppressed and cytogenetics analysis revealed trisomy 8. The patient was eligible for the AML17 trial with induction chemotherapy (daunorubicin and cytarabine). Bone marrow analysis at 21 days post chemotherapy showed that normal hematopoietic tissue was almost completely replaced by blast cells with large areas of hypocellularity (Fig. 1a, Supplementary Fig. 2). These findings indicated persistent AML and induction failure and the patient received 1 cycle of FLAG-Ida (fludarabine, cytarabine, idarubicin and G-CSF) on the high-risk arm of the AML17 trial. Bone marrow analyses 23 days post chemotherapy showed 60% blast cells and markedly suppressed granulopoiesis and hematopoiesis (Fig. 1a, Table 1). At this point the patient was removed from the AML17 trial, advised that his AML was persistent and refractory to salvage chemotherapy and discharged to palliative care.

**Table 1:** Bone marrow biopsy, blood test results, and clinical assessment from diagnosis, remission and relapse

Variable	Diagnosis	21 days post induction chemotherapy	23 days post salvage chemotherapy	2 months into ascorbate treatment	Relapse	Relapse post induction chemotherapy	Relapse	Relapse	Normal range
Timeline (months post diagnosis)	0	1	2	4	34	36	38	41	
Bone marrow blast count (% of nucleated cells)	75	30–80	57–65	0	76	>90	61–85	96	**<5**
Hemoglobin (g/dL)	9.8	11.2	9.7	13.3	11.0	9.6	12.1	9.9	**13.0–17.5**
White Cell Count (×10^9^/L)	1.53	1.08	0.26	3.2	1.6	1.6	2.1	3.6	**4.0–11.0**
Neutrophils (×10^9^/L)	0.14	0.46	0.0	1.8	0.4	0.29	1.3	0.36	**1.9–7.5**
Circulating blasts (×10^9^/L)	0.2	0.0	0.0	0.0	Occasional	0.04	0.0	2.4	**0**
Platelets (×10^9^/L)	73	228	16	83	89	89	116	36	**150–400**
Molecular Pathology^a^	*NPM1+*ve	ND^b^	ND	*NPM1*–ve	ND	ND	ND	ND	
Clinical assessment	Acute Myeloid Leukemia	Refractory to chemotherapy	Refractory to chemotherapy	Clinical remission	Relapse	Refractory to chemotherapy	Persistent AML	Persistent AML	

^a^Molecular Pathology testing involved PCR and Sanger sequencing in order to detect mutations in *NPM1*, *CEPBA* and *FLT3*

^b^ND, investigation was not done at this time point

**Fig. 1:**
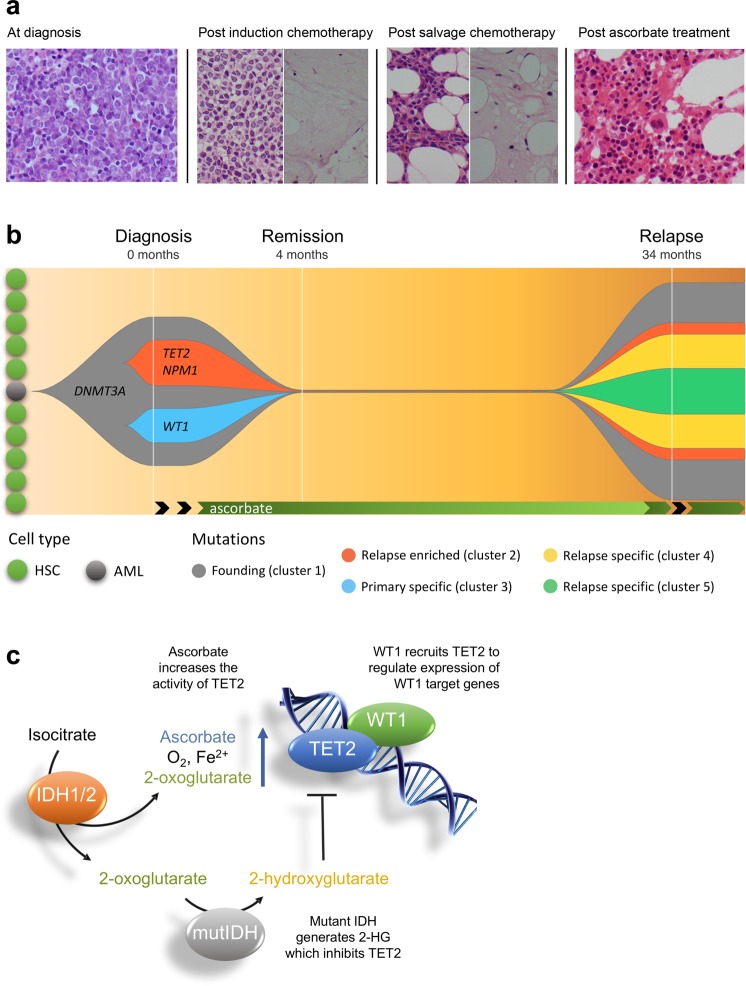
Bone marrow biopsies, clonal evolution, and proposed mechanism for the effect of ascorbate in TET2 compromised AML. **a** Bone marrow biopsies from the patient before and after treatment. At diagnosis, approximately 76% of total nucleated cells were blast cells. At 21 days following induction chemotherapy, trephine biopsy showed that normal hematopoietic tissue was almost completely replaced by blast cells (up to 80% of the total number of cells). In addition, there were areas of hypocellularity and necrosis. After salvage chemotherapy, there was persistent suppression of granulopoiesis with up to 65% blast cells and large areas of necrosis. Two months following ascorbate treatment, a bone marrow biopsy showed robust evidence of tri-lineage hematopoiesis and no blast cells, consistent with morphological remission. See Supplementary Fig. 2 for more images. **b** Clonal evolution of AML in this patient. The data used to generate this plot can be found in Supplementary Table 1. At least three major clusters were present in the diagnostic bone marrow. On the basis of variant allele frequency (VAF), the founding clone (cluster 1) contained somatic mutations in *DNMT3A* and eight other genes (*STAT5B*, *EEF1A2*, *CLCN2*, *KCND3*, *ATP2C1*, *CFLAR*, *PALB2*, *FAT3*). Subsequently, one subclone developed mutations in *TET2*, *NPM1*, and *TAF2* (cluster 2) with a separate subclone developing mutations in *WT1, ALDH16A1*, and *FAM8A1* (cluster 3). Whole exome sequencing post treatment with ascorbate did not detect any variants. At relapse, two of the three clones reemerged (clusters 1 and 2), with the addition of nine new mutations. The VAFs of the *DEFA5* and *MYC* mutation place them within the *TET2* subclone (cluster 4). Mutations in, *ABCA1*, *PPM1E* and *TRIM29* (cluster 5) could either fall within, or outside, of the *TET2* sublcone. The VAFs of *RNF40*, *MTDH*, *DST* and *PBLD* make them harder to place within the clonal structure and are not included here for clarity. This is potentially due to loss of heterozygosity which is supported by the fact that there are now VAFs greater than 50%. Black arrows indicate treatment with chemotherapy. HSC, hematopoietic stem cell. AML, acute myeloid leukemia stem cell. **c** Proposed mechanism for effect of ascorbate where mutations affect the *IDH1/2*-*TET2*-*WT1* pathway. Loss of function mutations in *WT1* or *TET2* and gain of function mutations in *IDH1* or *IDH2*, are early changes in the development of AML. These mutations are mutually exclusive in AML and all lead to decreased TET2 activity (see Supplementary Fig. 1) in 30–50% of AML. The TET enzymes are dependent on ascorbate for optimal activity, and providing additional ascorbate has been shown to increase the activity of TET2. Therefore, we propose that the benefit of ascorbate might extend to any mutation that affects the *IDH1/2*-*TET2*-*WT1* pathway. MutIDH, Mutant *IDH1* or *IDH2*. 2-HG, 2-hydroxyglutarate

One week after leaving the hospital, the patient commenced intravenous ascorbate treatment twice a week at a GP clinic (Ascor L 500®). The dose was gradually increased from 35 g to 95 g over approximately one month. Treatment with intravenous ascorbate resulted in clinically observed improvement which prompted repeat bone marrow testing. Blood film analyses revealed normal granulocyte and lymphocyte morphology, with mild suppression of granulopoiesis. Blast cells were not visible in the bone marrow, and trisomy 8 was not detected. These changes, along with tri-lineage proliferation and differentiation, were consistent with clinical remission (Fig. 1a, Table 1 and Supplementary Fig. 2). Ascorbate treatment was continued twice-weekly for the first year, fortnightly for the second year and once every 3–4 weeks in the third year after establishing clinical remission. Clinical remission was maintained for 2.5 years at which point relapse occurred (Table 1). Bone marrow analysis showed a return of AML (76% blasts, trisomy 8). The patient passed away approximately ten months after the recurrence of AML. See supplementary information for full details of the clinical timeline.

To investigate whether the reported clinical response to ascorbate treatment was associated with mutations in the *IDH1/2*-*TET2*-*WT1* pathway, we performed whole exome sequencing (WES) of blood and bone marrow samples from this patient at diagnosis, remission and relapse (see Supplementary Information). WES of DNA obtained from bone marrow cells at diagnosis revealed mutations in four genes known to drive the development of AML (*DNMT3A*, *TET2*, *WT1* and *NPM1*), along with 11 mutations in other genes predicted to have a moderate effect on protein function (Supplementary Table 1). None of these mutations were detected in the remission sample. At relapse, the same *DNMT3A*, *TET2*, and *NPM1* variants seen at diagnosis were present, along with 7 of the other 11 original mutations. In addition to this, 9 new mutations were also detected (Supplementary Table 1). Notably, the *WT1* variant was absent at relapse.

We investigated the potential impacts of these mutations on protein function. The heterozygous *DNMT3A* mutation found in the patient results in a leucine to valine substitution at position 295 and it is likely that protein function is disrupted, including DNA/histone binding as well as other protein interactions (Supplementary Fig. 3). The single base substitution may also introduce an alternative splice site and available evidence suggests likely pathogenicity. The 20 bp deletion in *TET2* found in this patient occurs in the last coding exon (Supplementary Fig. S4A). The deletion creates a premature stop codon (p.Leu1837fs), resulting in loss of the last 179 amino acids of TET2, including one of the catalytic site residues that coordinates binding to methylcytosine (H1904) and residues that bind iron and zinc (H1881 and H1912). For structural overlays and relevant references please see Supplementary Fig. S4B. Interestingly, functional assays have shown that the minimum sequence required for TET2 activity is 1129–1936^15^. Collectively, this data strongly suggests that the deletion found in this patient will result in a loss of TET2 activity of the affected allele. The *WT1* mutation also results in a premature stop codon and truncated protein with serious disruption of protein function and likely pathogenicity (Supplementary Fig. S5). The *NPM1* mutation was an insertion of CCGG in exon 12 (type Km) known to affect the function of this protein and to be pathogenic in AML.

Based on this information, and the variant allele frequencies (VAFs) of the mutations, we have illustrated the clonal evolution of AML in this patient (Fig. 1b). The *DNMT3A* mutation likely occurred earliest, which is consistent with data from large cohorts^2^. Subsequently, the *TET2* and *WT1* mutations appear to have arisen in separate clones. While the VAF of these variants allows for the possibility that the *TET2* and the *WT1* mutations were present in same subclone, the fact that the *WT1* and two other variants were not present at relapse suggests otherwise. Furthermore, the co-occurrence of both mutations in the same clone would be functionally redundant, and their mutual exclusivity in large cohorts supports their existence in two separate clones^6,7^. Finally, the VAF of the *NPM1* variant tracks closely with the *TET2* variant and is likely to be present in the same subclone.

Pre-clinical evidence suggests that ascorbate could be beneficial for patients with AML harboring mutations in *TET2* and *IDH1/2* with restoration of the residual TET2 activity providing a likely mechanism^10–13^. Although the effect of ascorbate in models of AML with *WT1* mutations has not been explored, there are decreased levels of 5hmC in patients with mutant *WT1*, indicative of reduced TET2 activity^6,7^. Therefore, we propose that ascorbate could have potential as a treatment option in AML where mutations affect the *IDH1/2*-*TET2*-*WT1* pathway (Fig. 1c, Supplementary Fig. 1b). The WES from our patient, which revealed that two of the genes in this pathway, *TET2* and *WT1*, were mutated in separate clones, is consistent with this hypothesis. The *WT1* mutant clone did not re-emerge at relapse, suggesting that it was more sensitive to treatment. In contrast to *WT1*, the *DNMT3A*, *TET2* and *NPM1* mutant clones were present at relapse and therefore present below the level of detection during remission. It is likely that the additional mutations found at relapse contributed to the relapse, and provide a plausible explanation for the subsequent resistance to ascorbate treatment.

To date, ascorbate has shown potential as an adjunct treatment for AML patients receiving hypomethylating agent therapy^14^. Together with pre-clinical evidence^10–13^, our findings are consistent with the hypothesis that ascorbate could provide benefit as an adjunct treatment where mutations affect the *IDH1/2*-*TET2*-*WT1* pathway. Clinical trials explicitly designed to address this possibility are required and the insights provided by this case study will assist in the design of these studies.

## Supplementary information

Supplementary Information
