# Supplementary material for: Clinical remission following ascorbate treatment in a case of acute myeloid leukemia with mutations in TET2 and WT1
Source: Blood Cancer J. 2019 Oct 2;9(10):82. doi: 10.1038/s41408-019-0242-4 (PMC6775073; doi:10.1038/s41408-019-0242-4)
Supplement: Supplementary file 1 — Supplementary Information [file 41408_2019_242_MOESM1_ESM.docx]

Supplementary Information

Clinical remission following ascorbate treatment in a case of acute myeloid leukemia with mutations in TET2 and WT1

Andrew B. Das, Purvi M. Kakadia, Damian Wojcik, Lucy Pemberton, Peter J. Browett, Stefan K. Bohlander, Margreet CM Vissers

Table of Contents

Supplementary Findings page 3

Supplementary Methods page 6

Supplementary Table 1 page 9

Supplementary Figure 1 page 10

Supplementary Figure 2 page 11

Supplementary Figure 3 page 12

Supplementary Figure 4 page 14

Supplementary Figure 4 page 15

Supplementary References page 16

**Supplementary findings**

**Presentation.** A 42-year-old male presented to his GP with swelling in the right inguinal region, fever and rigors. Full blood count revealed pancytopenia, and the presence of some blasts raised the suspicion of AML or high-grade lymphoma. On admission to hospital he had no B symptoms, no palpable lymphadenopathy and no signs of bleeding. Full blood count showed moderately severe pancytopenia (Table 1, Diagnosis). A bone marrow aspirate revealed markedly hypercellular trails packed with medium sized blasts accounting for approximately 75% of total nucleated cells (Fig. 1A). Granulopoiesis was markedly suppressed and 25% of nucleated cells stained positive for MPO. Cytogenetics analysis revealed trisomy 8. Molecular hematology found no evidence for *CEBPA*, *FLT3*-ITD or *FLT3*-TKD mutations, but did reveal an *NPM1* Type Km mutation with an insertion of CCGG in exon 12. Collectively, these findings confirmed diagnosis of AML, and the patient was offered treatment with intensive chemotherapy.

**Induction and salvage chemotherapy.** The patient was eligible for the AML17 trial and commenced induction chemotherapy (daunorubicin and cytarabine). In a bone marrow aspirate and trephine biopsy carried out 21 days post chemotherapy, blast cells were found to account for 30-80% of nucleated cells in the aspirate (Table 1). Trephine biopsy showed that normal hematopoietic tissue was almost completely replaced by blast cells, and in addition, there were large areas of hypocellularity and apparent bone marrow failure (Fig. 1B, Supplementary Fig 2). These findings are consistent with persistent AML and induction failure. The patient was then moved to the high-risk arm of the AML17 trial where he received 1 cycle of FLAG-Ida (fludarabine, cytarabine, idarubicin and G-CSF). Bone marrow analyses were performed 23 days post chemotherapy. Although there was some regeneration, granulopoiesis was still suppressed and other lineages were markedly suppressed. Blast cells were present at 60% (Fig. 1C and Table 1). At this point the patient was removed from the AML17 trial, and advised that his AML was persistent and refractory to salvage chemotherapy.

**Ascorbate treatment.** One week after leaving the hospital, the patient commenced intravenous ascorbate treatment twice a week at a GP clinic. The dose was gradually increased from 35g to 95g over approximately one month. A dose of 95g twice a week was maintained for approximately one year, after which it was reduced to 95g fortnightly. In the first months following initiation of ascorbate treatment the patient was seen in clinic by the consultant hematologist and regular blood tests revealed improved blood cell counts. Therefore, bone marrow analyses were undertaken again at 10 weeks post initiation of ascorbate treatment (Table 1). Blood films revealed normal granulocyte and lymphocyte morphology. While the bone marrow aspirate indicated mild suppression of granulopoiesis, blast cells were not visible, which, along with tri-lineage proliferation and differentiation, was consistent with morphological remission (Fig. 1D, Table 1 and Supplementary Fig. 2). No mutations in *FLT3*, *CEPBA* or *NPM1* were detected. These findings were interpreted as clinical remission.

**Remission.** One month after remission was confirmed by bone marrow aspirate, the patient underwent interim consolidation chemotherapy (intermediate dose Ara-C) but for personal reasons declined to proceed with ablative conditioning and allogeneic stem cell transplant. Ascorbate treatments were temporarily stopped during chemotherapy and resumed immediately after. Ascorbate infusions were continued twice weekly for year, at which point they were decreased to fortnightly. During this period, the patient returned to full-time work and resumed regular sporting activities. Blood counts were all within the normal range except platelets, which reached 136-144×10^9^/L (ref 150-400×10^9^/L). After 2 years, ascorbate treatment decreased to once every 2-3 weeks and regular blood tests continued during this time.

**Relapse.** Approximately three years after the initial presentation the patient contracted a respiratory illness and found it hard to concentrate at work. A full blood count revealed pancytopenia (Table 1, Relapse). Subsequent bone marrow analysis showed a return of AML (76% blasts, trisomy 8). Idarubicin and Ara-C were commenced as induction therapy. Repeat bone marrow biopsies one month later showed aggressive progression of the disease (>90% blast cells) and the patient was advised that the relapsed AML was refractory to chemotherapy. After hospital discharge ascorbate treatments were resumed with twice weekly infusions of 95g and this regime was maintained for a further six months. Over this time, he reported feeling much better, resumed sporting activities and returned EORTC Core 30 Global QOL scores around 80% for a period of 21 weeks. Improving blood counts prompted a follow-up bone marrow biopsy at two months post chemotherapy which revealed persistent AML (61-85% blasts). Eventually, his blood counts dropped off and a final bone marrow biopsy confirmed the same assessment (Table 1). The patient passed away approximately ten months after the recurrence of AML.

**Supplementary materials and methods**

**Patient specimens.** To investigate the potential correlation between treatment and AML subtype, whole exome sequencing (WES) was carried out on DNA isolated from the patient's bone marrow (BM) samples at diagnosis, remission and relapse. DNA extraction, WES and data analysis were performed using the methods reported previously^1^. Briefly, somatic variants were identified by comparing sequence reads from these three time points as well as annotating the variants with information from SNP databases. See below for further details. Ethics approval for this study was obtained from the Health and Disability Ethics Committee, NZ (reference 17/NTA/208) and informed consent was obtained from the patient’s family representative. All experimental procedures and methods were performed and carried out according to the guidelines and regulations of the Auckland District Health Board, the University of Auckland and the university of Otago.

**Whole exome sequencing.** Bone marrow genomic DNA (500 ng each) were sheared using the EpiShear™ Multi-Sample Sonicator (Active Motif). The condition used to obtain fragments in the size range of 100-400 bp was 65% amplitude, 3 rounds of 20 minutes with alternating 30 sec on and 30 sec off (total 30 minutes on time). Before each cycle, the temperature of the sonicator was ensured to be at 4°C. The quantity and the fragment size of the sheared DNA was assessed on a Tapestation 2200 (Agilent) with the high sensitivity D1000 tape. 100 ng of sheared DNA was used for the preparation of the whole exome libraries (WEL). WELs were prepared using the SureSelect XT2 (SSXT2) reagent kit and the SureSelect Clinical Research Exome V2 exome enrichment kit (Design ID #S06588914) following the manufacturer’s instructions (Agilent Technologies). The WEL were sequenced on a NextSeq500 (NCS v2.0, Illumina Inc.) to obtain around 40 to 44 million paired end reads (2x150 bp) per exome.

**Data analysis**. The quality of the sequences was assessed using Fastqc (https://www.bioinformatics.babraham.ac.uk/projects/fastqc/). The sequences were aligned to the human reference genome (hg19) with BWA (bwa 0.7.12)^24^. The resulting sam files were converted to bam files and then the bam files were sorted using Samtools (Samtools-1.3.1)^25^. Mpileup files were generated (Samtools 1.3.1) with the following parameters: maximum depth (-d) 500, minimum base quality (-Q) 15 and minimum mapping quality (-q) 10. In order to call the somatic variants in the samples at diagnosis and relapse, the somatic function of Varscan v2.3.9^26^ was used with germline sample being the BM in remission to generate VCF (variant call format) files^27^. The somatic variants in the vcf files were annotated with information from various SNP databases (dbSNP138 etc) using ANNOVAR^28^ followed by the annotation for the variants’ effect with SnpEffect^29^. Somatic or loss of heterozygosity variants predicted to have a 'High' (e.g. non-sense) or 'Moderate' (missense) impact and with a somatic p value of ≤0.001 were selected using SnpSift^30^. Variants present in dbSNP (142) or the 1000genome_Oct2014 database were excluded.

**WES Data Quality Assessment**. To assess the quality of the data and efficiency of the target enrichment procedure, hybrid selection, per target coverage, insert size, alignment summary and GC bias metrics were generated using picard-tools-2.4.1 (http://picard.sourceforge.net).

**Data availability.** Fig 2a is based on the data provided in Supplementary Table 1. The full WES dataset generated during the current study is available from the corresponding author on reasonable request. All data provided is anonymized to respect the privacy of patient. Any clinical information not provided in the manuscript may be subject to patient confidentiality.


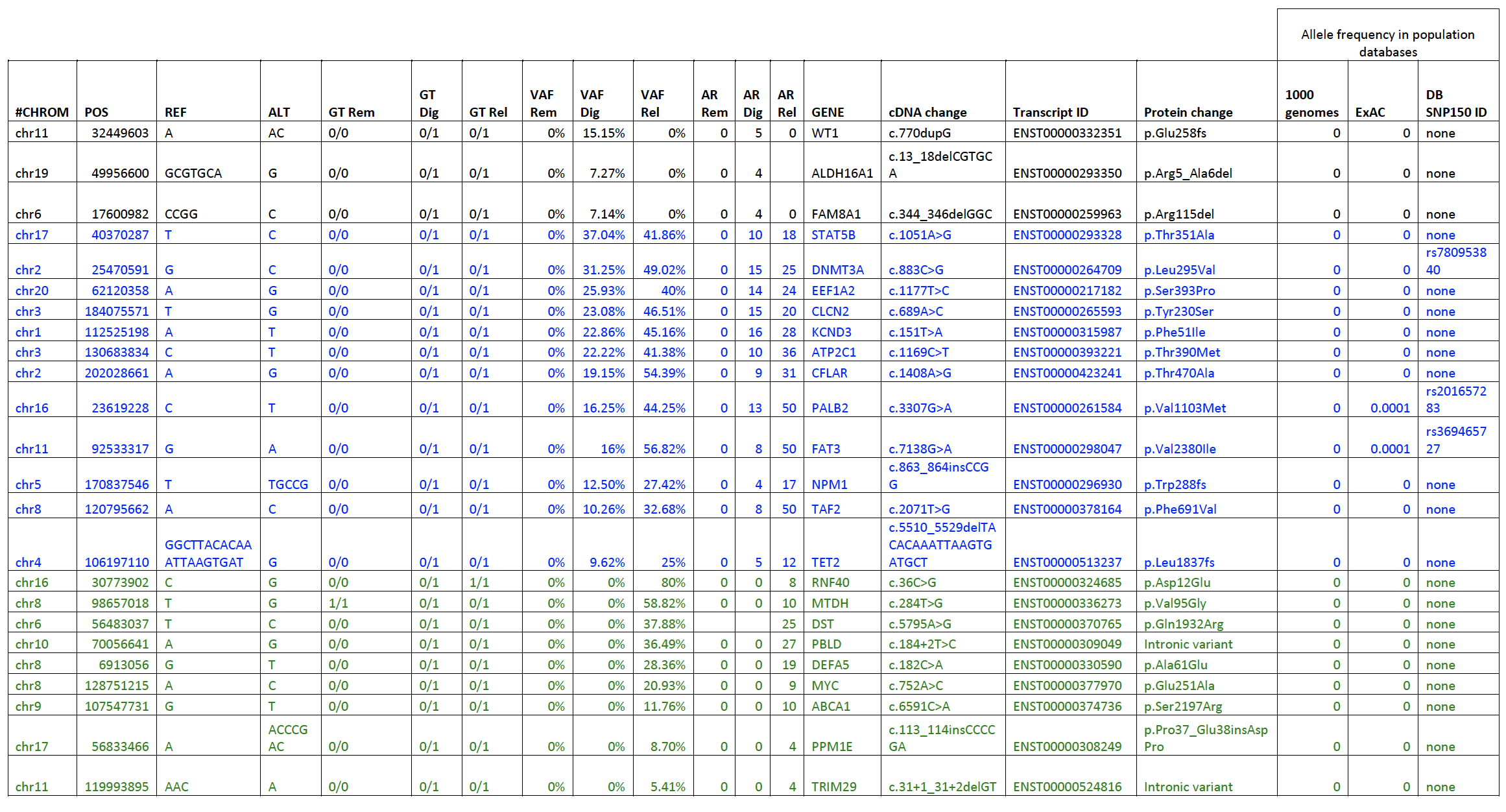


**Supplementary Table 1. Summary of variants found at diagnosis and relapse compared to remission.**

WES and analysis of data was carried out on DNA from the patient at diagnosis, relapse, and remission as previously described^1^. The somatic variants in the vcf files were annotated with information from various SNP databases (dbSNP138 etc) using ANNOVAR^2^ followed by the annotation for the variants’ effect with SnpEffect^3^. Somatic or loss of heterozygosity variants predicted to have a 'High' (e.g. non-sense) or 'Moderate' (missense) impact and with a somatic p value of ≤0.001 were selected using SnpSift^4^. The variants found at diagnosis only are detailed in **black text**, variants persisting at relapse are detailed in **blue text** and variants arising at relapse only are detailed in **green text**.

**
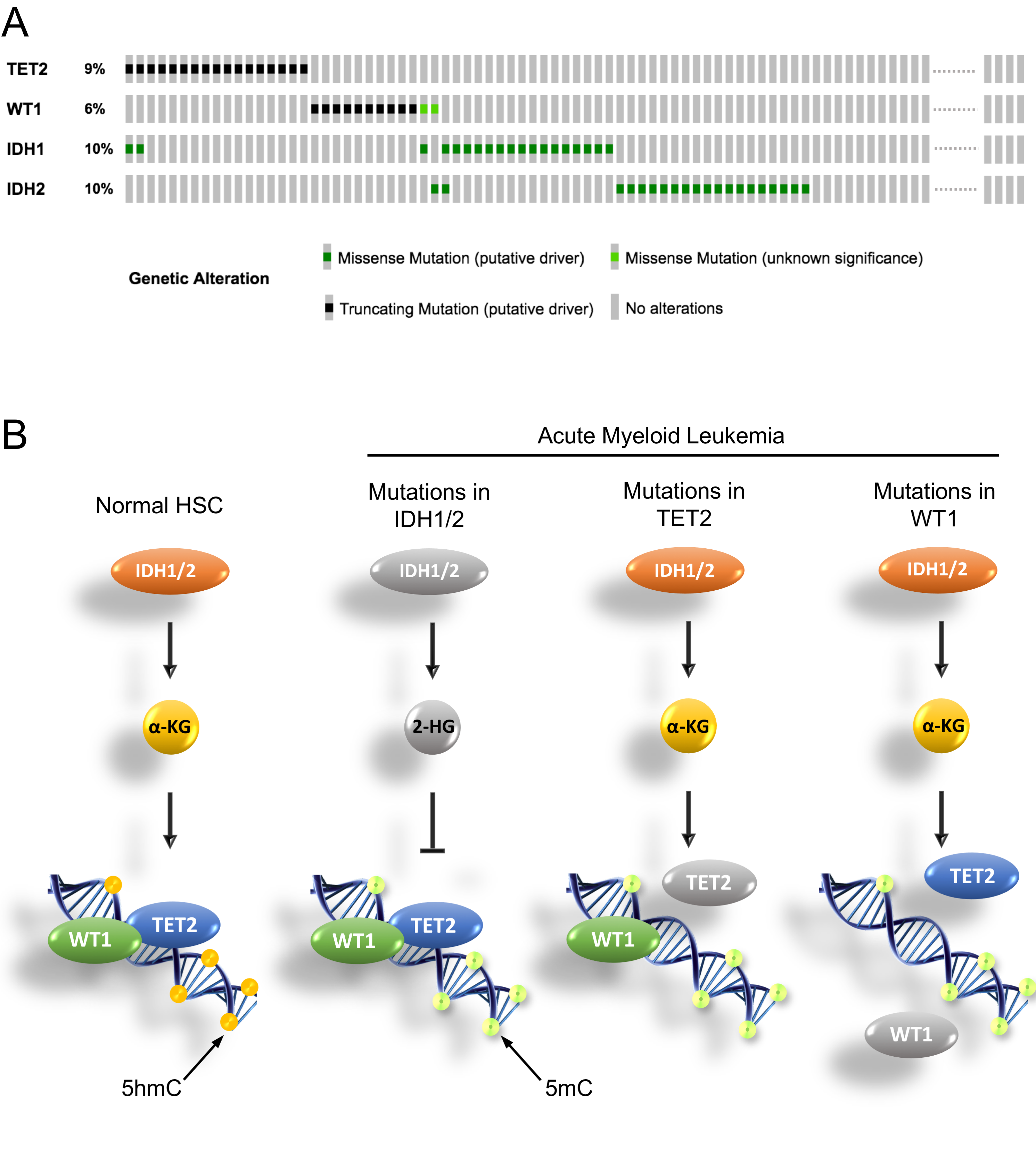
**

**Supplementary Figure 1. *IDH1*, *IDH2*, *TET2* and *WT1* mutations in AML**

**A, Mutations in *IDH1*, *IDH2*, *TET2* and *WT1* are mutually exclusive in AML.** TCGA data for 200 AML patients was analysed and visualised using cBioPortal^5,6^. Each column represents an individual patient, with respective mutations marked as indicated on the figure. With a few exceptions, mutations in these four genes generally do not occur in the same patient. Collectively, 63 patients out of 200 (31.5%) have mutations in at least one of these genes. Patients 75-196 have no mutations in the genes queried and have been replaced by the grey dots for clarity. HSC, hematopoietic stem cell. 5hmC = 5-hydroxymethylcytosine. 5mC, 5-methylcytosine. Other studies have found the collective mutation rates to be between 30-50%^7–10^.

**B, *IDH1/2-TET2-WT1* constitutes a pathway that suppresses leukemogenesis.** *TET2* activity is decreased in patients with mutations in *TET2*, *IDH1*, *IDH2* and *WT1*^11–13^. Mutations in *IDH1* and *IDH2* generate the oncometabolite 2-hydroxyglutarate which inhibits TET2^11^. On the other hand, WT1 is a transcription factor that binds directly to TET2 and recruits it to DNA^9^. Together with their mutual exclusivity, the evidence suggests that mutations affecting the function of these proteins comprise distinct subtype of AML characterized by dysregulated DNA (hydroxy)methylation^12^. This figure was adapted from the graphical abstract of Rampal et al^12^.


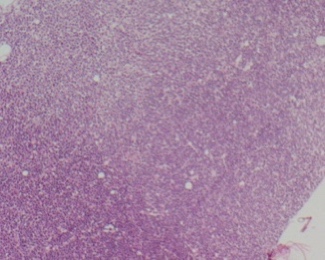

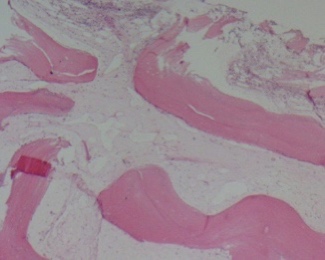

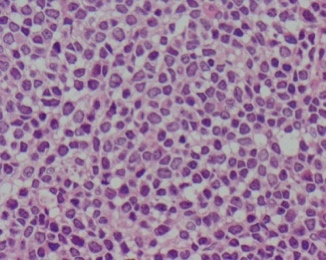

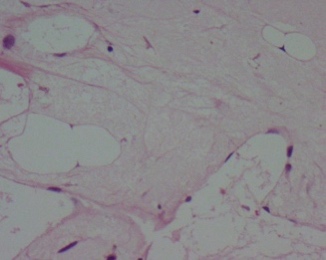

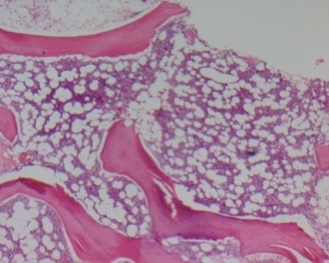

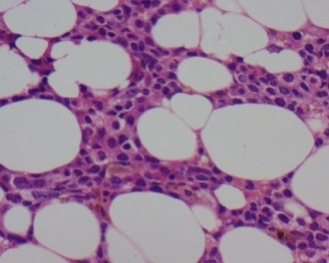

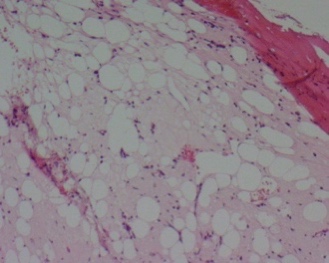

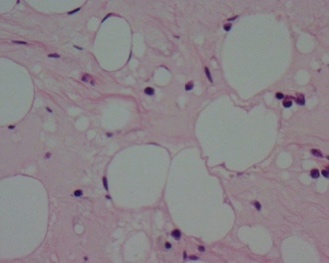

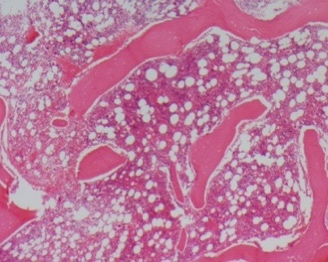

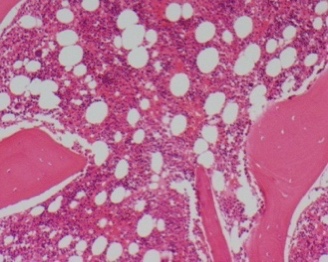

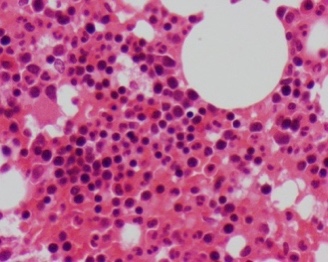

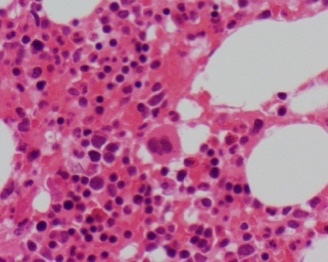


Post-induction chemotherapy

Post salvage chemotherapy

Two months into ascorbate treatment

**Supplementary Figure 2. Additional Imaged from bone marrow biopsy**Additional images of bone marrow biopsies from the patient before and after treatment, at lower and higher magnification (top and bottom rows of images, respectively). Following induction chemotherapy, normal hematopoietic tissue was almost completely replaced by up to 80% blast cells, with areas of hypocellularity and necrosis. After salvage chemotherapy, there was persistent suppression of granulopoiesis with up to 65% blast cells and large areas of necrosis. Ten weeks following ascorbate treatment, bone marrow showed robust evidence of tri-lineage hematopoiesis and no blast cells, consistent with morphological remission.

**
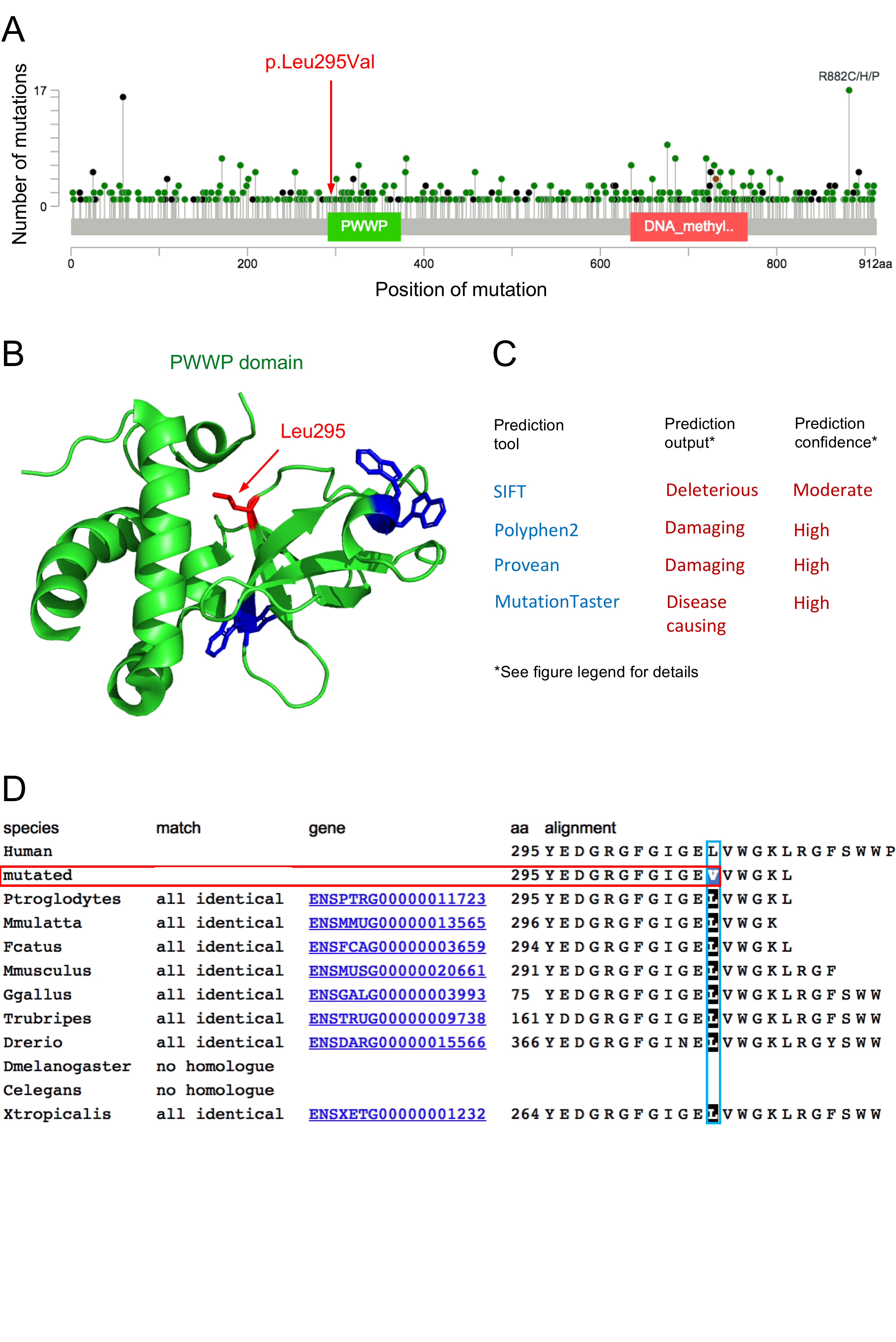
**

**Supplementary Figure 3. Functional impact of the *DNMT3A* substitution** (see next page for legend).

**Legend for Supplementary Figure 3.**

**A, Position of mutation found in the patient.** Graph showing 1145 mutations in *DNMT3a* across 233 studies collated by cBioPortal^5,6^. Green pins represent missense mutations and black pins represent truncation mutations. A heterozygous single base substitution in *DNMT3* (c.883C>G; p.Leu295Val; ENST00000264709) was found in this patient. The leucine to valine transition falls within the PWWP domain which is crucial for DNMT3A binding to DNA and modified histones^14^.

**B, 3D structure of the PWWP domain.** The relative positions of Leu295 and tryptophan (Trp-Trp) are shown in red and blue respectively. This structure was produced with PyMol (The PyMOL Molecular Graphics System, Version 2.2.0 Schrödinger, LLC) using the pdb file 3LLR^14^ downloaded from www.rscb.org.

**C, Predicting the functional impact of the variant**. The following algorithms were used to predict potential functional impacts of the substitution in *DNMT3A*: SIFT^15^, Polyphen2^16^, Provean^17^, and MutationTaster^18^. SIFT uses multiple sequence alignment to generate predictions. For this variant, SIFT score was 0.00 (deleterious if <0.05) and a median conservation value of 3.41 (interpret with caution because the sequences used for alignment were highly similar). The more diverse the sequences available for alignment, the higher the confidence that a prediction is correct, because this makes it more likely that evolution has had time to conserve the residue. Polyphen2 uses a combination of sequence alignment and 3D structural changes, and returned a score of 1 (scale 0 to 1, with 1 being most likely damaging). Provean uses an alignment based approach and had an output score for this variant of 2.41. Using a threshold of 2.41 for likely damaging, the sensitivity and specificity for this prediction is approximately 81% and 78% respectively. MutationTaster combines a wide range of bioinformatic tests including sequence alignment, conservation across species, introduction of splice sites, position of mutation within protein and combines these scores to provide a prediction with an associated confidence (0-1). For this variant, MutationTaster predicted it to be disease causing with a probability of 0.99999986. In addition to affecting the PWWP domain, this variant was also predicted to affect interaction with DNMT3B and DNMT1. Interestingly, Mutationtaster also indicated that this change introduces an alternative splicing site potentially leading to a shortening of the protein. Taken together, it is likely that DNMT3A function is disrupted in this patient, but experimental validation would be required for certainty.

**D, Conservation of Leu295.** Leucine at position 295 is conserved across all species for which there are homologues. Graphic adapted from MutationTaster output.

**
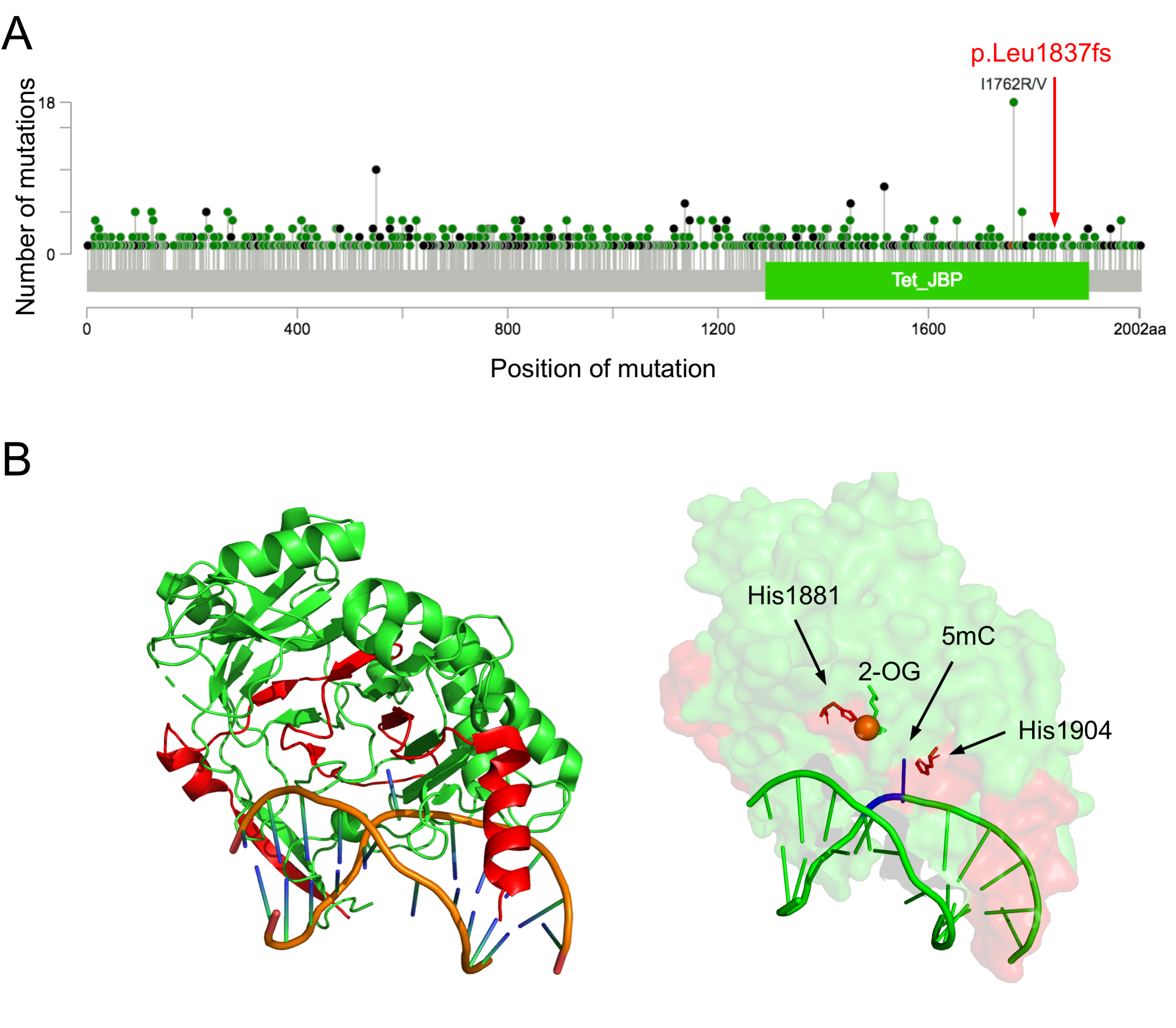
**

**Supplementary Figure 4. Functional impact of the *TET2* deletion.**

**A, Position of mutation found in the patient.** Graph showing 1480 mutations in *TET2* across 233 studies collated by cBioPortal^5,6^. Green pins represent missense mutations and black pins represent truncation mutations. The 20 bp deletion in *TET2* (c.5510_5529delTACACAAATTAAGTGATGCT; p.Leu1837fs; ENST00000513237) found in this patient occurs in the last coding exon (exon 11). Previous studies have found that ~30% of *TET2* mutations occur in this exon in myeloid malignancies^10,19^. The deletion creates a premature stop codon, which results in a loss of the last 179 amino acids (out of 2002) of TET2.

**B, Structure of the affected domain and functional impact.** The premature stop codon disrupts the dioxygenase domain (TET_JBP) which binds to DNA and oxidises methylcytosine to cytosine. **Left panel,** 3D structure of the domain of interest in green (sequence retained) and red (sequence lost as a result of truncation). DNA is shown in orange and blue. **Right panel,** protein surface is shown with transparency, and DNA in green and blue. The sequence lost due to truncation includes one of the catalytic site residues (H1904) that coordinates binding to methylcytosine (5mC)^20^. Residues that bind iron and zinc (H1881 and H1912) are also lost. Interestingly, previous functional assays have shown that the minimum sequence required for activity is 1129-1936^20^. Collectively, this data strongly suggests that the deletion found in this patient will result in a loss of TET2 activity of the affected allele. These structures were produced with PyMol (The PyMOL Molecular Graphics System, Version 2.2.0 Schrödinger, LLC) using the pdb file 4NM6 downloaded from www.rscb.org. 2-OG, 2-oxoglutarate.


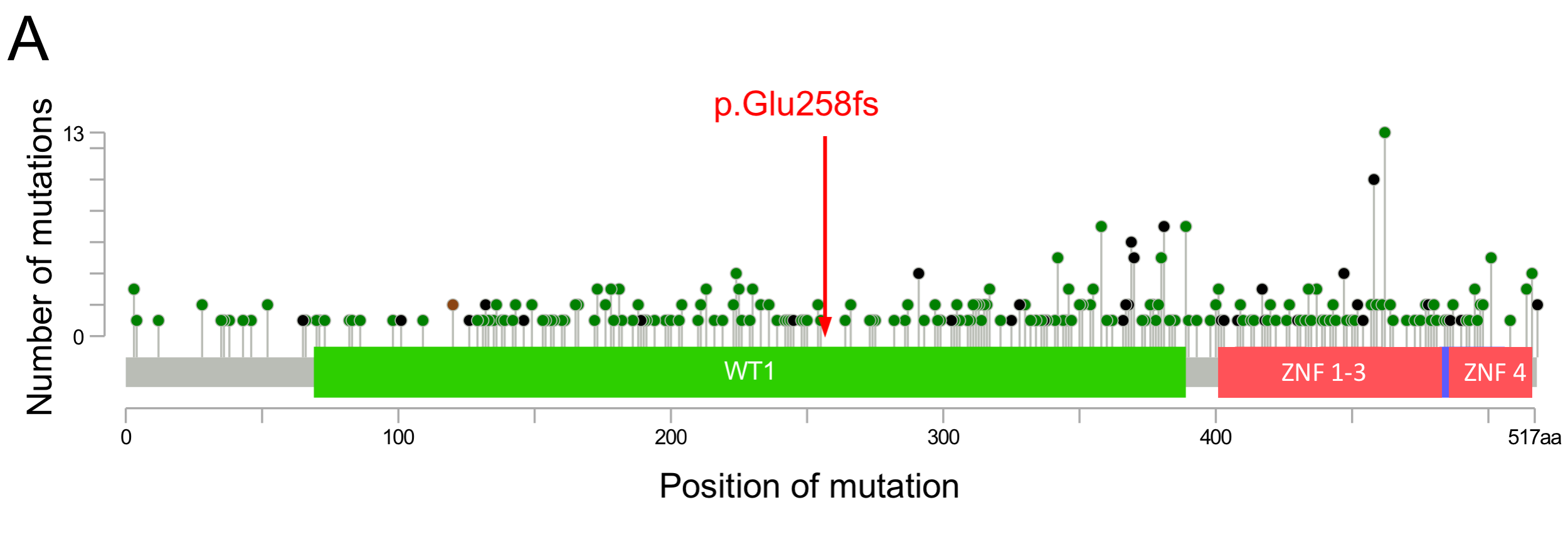


**Supplementary Figure 5. Functional impact of the *WT1* insertion.** Graph showing 614 mutations in *WT1* across 233 studies collated by cBioPortal^5,6^. Green pins represent missense mutations and black pins represent truncation mutations. The heterozygous single base insertion found in this patient in *WT1* (c.770dupG; p.Glu258fs; ENST00000332351) creates an immediate stop codon, truncating the protein by 260 amino acids. This includes the four zinc-finger domains (ZNF 1-3 and ZNF4) which are required for binding to DNA^21^, impairing the function of WT1 from one allele. Interestingly, WT1 is a crucial binding partner required to target TET2 to DNA appropriately^9^, the hydroxymethylation associated with activation of WT1 target genes requires TET2 activity. Importantly, the loss of WT1 or TET2 in AML results in a loss of hydroxymethylation and concurrent loss of expression of WT1 target genes^12^. Wilms-tumour 1, is a transcription factor with 4 zinc finger domains. Zinc fingers 2-4 recognise the specific sequence 5'-GCG(T/G)GGGCG-3' and mediate the binding of WT1 to DNA^21^. The first zinc finger domain may mediate further sequence specificity^22^. There are four different transcripts expressed in the haematopoietic lineage, based on the possible combinations of two splicing events. The first involves the exclusion of exon 5 (17 nucleotides) and the second involves insertion of three amino acids between zinc-finger 3 and 4 (shown in blue). WT1 without the 3 amino acids, lysine, threonine and serine (-KTS) recognises the DNA consensus sequence, whereas the form with the insertion (+KTS) is likely to be involved in mRNA splicing^23^. The relative ratio of the +KTS/-KTS isoforms can affect differentiation and quiescence in haemopoietic stem cells and myeloid progenitors^24^. Collectively, this data strongly suggests that the deletion found in this patient will result in a loss of WT1 activity of the affected allele.

**Supplementary references**

1 Kakadia PM, Van de Water N, Browett PJ, Bohlander SK. Efficient identification of somatic mutations in acute myeloid leukaemia using whole exome sequencing of fingernail derived DNA as germline control. *Sci Rep* 2018; **8**: 13751.

2 Wang K, Li M, Hakonarson H. ANNOVAR: Functional annotation of genetic variants from high-throughput sequencing data. *Nucleic Acids Res* 2010; **38**: 1–7.

3 Cingolani P *et al.* A program for annotating and predicting the effects of single nucleotide polymorphisms, SnpEff: SNPs in the genome of Drosophila melanogaster strain w1118; iso-2; iso-3. *Fly (Austin)* 2012; **6**: 80–92.

4 Cingolani P *et al.* Using Drosophila melanogaster as a model for genotoxic chemical mutational studies with a new program, SnpSift. *Front Genet* 2012; **3**: 1–9.

5 Cerami E *et al.* The cBio Cancer Genomics Portal: An open platform for exploring multidimensional cancer genomics data. *Cancer Discov* 2012; **2**: 401–404.

6 Gao J *et al.* Integrative Analysis of Complex Cancer Genomics and Clinical Profiles Using the cBioPortal Complementary Data Sources and Analysis Options. *Sci Signal* 2014; **6**: 1–20.

7 Metzeler KH *et al.* Spectrum and prognostic relevance of driver gene mutations in acute myeloid leukemia. *Blood* 2016; **128**: 686–698.

8 TCGARN. Genomic and Epigenomic Landscapes of Adult De Novo Acute Myeloid Leukemia. *N Engl J Med* 2013; **368**: 2059–74.

9 Wang Y *et al.* WT1 recruits TET2 to regulate its target gene expression and suppress leukemia cell proliferation. *Mol Cell* 2015; **57**: 662–673.

10 Papaemmanuil E *et al.* Genomic Classification and Prognosis in Acute Myeloid Leukemia. *N Engl J Med* 2016; **374**: 2209–2221.

11 Figueroa ME *et al.* Leukemic IDH1 and IDH2 Mutations Result in a Hypermethylation Phenotype, Disrupt TET2 Function, and Impair Hematopoietic Differentiation. *Cancer Cell* 2010; **18**: 553–567.

12 Rampal R *et al.* DNA Hydroxymethylation Profiling Reveals that WT1 Mutations Result in Loss of TET2 Function in Acute Myeloid Leukemia. *Cell Rep* 2014; **9**: 1841–1856.

13 Magotra M *et al.* Immunohistochemical loss of 5-hydroxymethylcytosine expression in acute myeloid leukaemia: relationship to somatic gene mutations affecting epigenetic pathways. *Histopathology* 2016; **69**: 1055–1065.

14 Wu H *et al.* Structural and histone binding ability characterizations of human PWWP domains. *PLoS One* 2011; **6**. doi:10.1371/journal.pone.0018919.

15 Sim NL *et al.* SIFT web server: Predicting effects of amino acid substitutions on proteins. *Nucleic Acids Res* 2012; **40**: 452–457.

16 Adzhubei I, Jordan DM, Sunyaev SR. *Predicting functional effect of human missense mutations using PolyPhen-2*. 2013 doi:10.1002/0471142905.hg0720s76.

17 Choi Y, Chan AP. PROVEAN web server: A tool to predict the functional effect of amino acid substitutions and indels. *Bioinformatics* 2015; **31**: 2745–2747.

18 Schwarz JM, Cooper DN, Schuelke M, Seelow D. Mutationtaster2: Mutation prediction for the deep-sequencing age. *Nat Methods* 2014; **11**: 361–362.

19 Moran-Crusio K *et al.* Tet2 Loss Leads to Increased Hematopoietic Stem Cell Self-Renewal and Myeloid Transformation. *Cancer Cell* 2011; **20**: 11–24.

20 Hu L *et al.* Crystal Structure of TET2-DNA Complex: Insight into TET-Mediated 5mC Oxidation. *Cell* 2013; **155**: 1545–1555.

21 Hamilton TB, Barilla KC, Romaniuk PJ. High affinity binding sites for the Wilms’ tumour suppressor protein WT1. *Nucleic Acids Res* 1995; **23**: 277–84.

22 Wang D *et al.* Role for first zinc finger of WT1 in DNA sequence specificity: Denys–Drash syndrome-associated WT1 mutant in ZF1 enhances affinity for a subset of WT1 binding sites. *Nucleic Acids Res* 2018; **46**: 3864–3877.

23 Markus MA *et al.* WT1 interacts with the splicing protein RBM4 and regulates its ability to modulate alternative splicing in vivo. *Exp Cell Res* 2006; **312**: 3379–3388.

24 Ellisen LW, Carlesso N, Cheng T, Scadden DT, Haber D a. The Wilms tumor suppressor WT1 directs stage-specific quiescence and differentiation of human hematopoietic progenitor cells. *EMBO J* 2001; **20**: 1897–1909.
